# How do overweight people dropout of a weight loss diet? A qualitative study

**DOI:** 10.1186/s40795-021-00480-w

**Published:** 2021-11-19

**Authors:** Leila Bazrafkan, Mohammad Amin Choobineh, Mehrnaz Shojaei, Alireza Bozorgi, Mohammad Hossein Sharifi

**Affiliations:** 1 Clinical Education Research Center, Shiraz University of Medical Sciences, Shiraz, Iran; 2 Department of Community Nutrition, School of Nutrition & Food Science, Isfahan University of Medical Sciences, Isfahan, Iran; 3 Research Center for Traditional Medicine and History of Medicine, Shiraz University of Medical Sciences, Shiraz, Iran; 4 Research Center for Traditional Medicine and History of Medicine, Shiraz University of Medical Sciences, Shiraz, Iran

**Keywords:** Overweight, Obesity, Dropout, Weight-loss diet, Qualitative study

## Abstract

**Background:**

The growing trend of overweight and obesity in many developed and developing countries in recent years has made obesity one of the most significant health problems in the world. The treatment of overweight and obese people is challenging, as patients have difficulty adhering to a weight-loss diet. Thus, the present study aimed to identify the reasons for the dropout of weight-loss diets.

**Methods:**

This qualitative study using content analysis was conducted in a comprehensive health center in Shiraz, southern Iran, between April and October 2020. The study was performed on 27 participants with a history of obesity and diet dropout selected via purposive and theoretical sampling. The data were gathered through semi-structured interviews and were thematically analyzed.

**Results:**

The participants included 25 females (92.6%) and two males (7.4%) with a mean age of 33.4 ± 8.4 years. Data analysis resulted in the emergence of three themes and 14 sub-themes. The first theme was personal reasons for diet dropout, which included six sub-themes; i.e., misunderstanding of diet, not having enough motivation, stress and hormonal disorder, having the feel of “being harmful to health”, lack of mental and psychological preparation, and personal taste. The second theme was familial and social reasons for diet dropout, including two sub-themes, i.e., social and familial problems. Finally, the third theme was the reasons related to diet characteristics, including six sub-themes: ineffectiveness of diet, expensiveness of diet food and dietary supplements, family problems, unavailability of food, unscientific and unconventional diets feeling bad about the diet, and unpalatable diet food. All the concepts were related to each other and resulted in a pattern revealing the experiences of overweight people and who had dropped out of weight-loss diets.

**Conclusion:**

The reasons for diet dropout were divided into three levels: personal reasons, familial and social reasons, and diet characteristics. Overall, clinicians should pay attention to the complexity of diets to increase the success rate of weight management. Based on the current study findings, a guideline is recommended to guide patients who dropout of weight-loss diets.

## Background

The growing trend of overweight and obesity in many developed and developing countries in recent years has made obesity one of the most significant health problems in the world [1, 2]. In Iran, the prevalence of overweight and obesity is rising due to urbanization and industrialization [3]. Evidence has revealed that obesity is linked to higher mortality rates from comorbidities such as Type 2 Diabetes (T2D), hypertension, dyslipidemia, obstructive sleep apnea, certain types of cancer, obstructive sleep apnea, polycystic ovarian syndrome, etc. infertility [4]. Approximately 2.5 million deaths occur each year as a result of obesity-related diseases. Moreover, studies have shown that the mortality risk associated with obesity has increased by 50–100% compared to the population with normal body mass indices [5].

The most common treatments for obesity include diet, exercise, and behavior therapy often referred to as lifestyle modifications [6, 7]. A significant number of weight-loss efforts have been repeatedly demonstrated to be a negative prognostic indicator of weight-loss success [8]. A Cochrane systematic review of 55 published interventions to manage obesity found the effectiveness of short-term interventions on reducing weight. However, these interventions showed small effects in the long run [9, 10]. To better understand why common therapies have had non-significant effects on weight loss, it is necessary to have detailed information, especially regarding behavioral, environmental, and psychosocial aspects. However, few questionnaires assess these aspects and the reasons for the dropout of weight-loss diets.

Dropout of weight-loss diet is one of the main reasons for therapy failure in obese patients, with the prevalence ranging from 10 to 80% in different studies [11, 12]. Dropout of weight-loss diet has been defined as attrition or failure to continue weight-loss treatment at any stage until the end of treatment [13]. Previous studies indicated that the influential factors in the dropout of a weight-loss diet included poor adherence, weight regains, inadequate and unsatisfactory weight loss, and high attrition levels [11, 12]. Scientific studies have also indicated that gender, age at the onset of obesity, unemployment, full-time employment, previous treatment experience, sedentary lifestyle, and body image could contribute to dropout of a weight-loss diet. However, limited studies have been conducted on this issue. No qualitative studies have assessed behavioral, environmental, and psychosocial aspects of dropout of weight-loss diets using reliable questionnaires.

Moreover, limited qualitative studies have been conducted on the reasons for the dropout of weight-loss diets worldwide. Qualitative studies have been performed on patients with metabolic diseases to determine the reasons for the dropout of weight-loss diets and the experiences of personals in diet programs. Thus, the present qualitative study aims to identify the reasons for the dropout of weight-loss diets.

## Method

### Design

This qualitative study was conducted following a content analysis approach using semi-structured interviews [14].

#### Setting

The present study was conducted in Shiraz University of Medical Sciences (SUMS), one of the public universities in Iran located in Shiraz. The university currently has 782 faculty members, more than 10,000 students, 200 majors, 54 research centers, 13 educational hospitals, and 17 faculties, including nutrition faculty. Several nutrition clinics affiliated to SUMS also provide services to patients and people who need nutritional services.

Participants and Sampling: This study was conducted in Shiraz, southern Iran, a comprehensive health center, between April and October 2020. The study’s inclusion criteria were aging above 18 years, being overweight or obese, having a history of receiving standard diets first, and diet dropout. Exclusion criteria were the history of severe gastrointestinal diseases, presence of systemic disorders, or mental diseases based on participants’ self-report, and in case of lack of cooperation at any stage of the study The participants were selected using purposeful sampling with maximum variation. Sampling was continued until data saturation [15].

The study data were collected using semi-structured interviews. The interview questions were focused on the participants’ experiences of going on diets for weight loss. The interviews were begun with such questions as “please describe your diet schedule,” “please talk about a successful diet schedule,” and “tell me about why you dropped out of your weight loss diet.” The interviews were digitally recorded and typed precisely in Microsoft Word software. The contents of the interviews were then reviewed and re-read several times to obtain a general understanding of the concepts. In this way, the main sentences and concepts of each line and each paragraph were defined and coded. The codes were identified based on the participants’ words or arguments. Following the extraction of the initial codes, they were merged and summarized. Afterward, the codes with the same concept and common characteristics and dimensions were included in one group.

#### Data analysis

Data analysis was done via thematic content analysis. In doing so, each interview was typed precisely in Microsoft Word software. The interview content was then reviewed and re-read several times to understand its concepts and was analyzed before the following interview. Hence, each interview determined the next person to be interviewed. The meaning units that consisted of words and sentences were abstracted and labeled with codes. Totally, 14 interviews were conducted in a single session, and four interviews were performed through two or three sessions. The coding procedure was iterative and was carried out line by line through comparative analysis under the following six stages of thematic analysis: [1] familiarizing with data, [2] generating initial codes, [3] searching for themes, [4] reviewing themes; various codes were compared based on the similarities and differences in meaning, [5] defining and naming themes, and [6] producing the report. Similar codes were grouped into subcategories, and then similar subcategories and domains were classified into major categories [16].

#### Trustworthiness

To verify scientific accuracy and trustworthiness, use was made of the four criteria proposed by Lincoln and Guba, i.e., credibility, transferability, consistency or dependability, and confirmability [14]. Credibility was determined via drowning in data, the dedication of time to collecting and interpreting the data, constant presence of the researcher in the clinic, preservation of raw data and records, keeping raw data documents, and keeping the documents of the researcher and the interviews. Transferability in qualitative studies implies that the findings can apply to other contexts or groups. This study attempted to provide a clear, precise, and purposeful description of the process and activities performed, including the procedures and findings (classes) to allow others to follow the study process and the characteristics of the study population. Sampling with maximum variation was yet another transferability technique utilized in this study. Consistency or dependability is similar to reliability in quantitative studies, which means that the data are consistent over time and under similar conditions. In this research, dependability was approved through the audit. In this way, the researcher extensively described all stages of the research from the beginning to the end so that external auditors could audit the documents. Confirmability indicates that the data are neutral and is approved when other researchers agree on the relationships or meanings of the data. To meet this criterion, qualitative researchers record their activities over time so that other researchers can follow them up. In this study, confirmability was achieved via other researchers’ evaluation of several interviews, texts, codes, and articles [17].

#### Ethical considerations

The Ethics Committee approved this study of SUMS (IR.SUMS.REC.1398.1299). In addition, the participants were ascertained about the confidentiality of their information, and their informed consent forms were obtained.

## Results

This study was conducted on 27 obese participants, including 25 females (92.6%) and two males (7.4%). The participants’ ages ranged from 18 to 56 years, with a mean age of 33.4 ± 8.4 years. Based on the results, 18 participants (66.7%) had a history of dropout of one weight-loss diet, and nine participants (33.3%) had a history of dropout of more weight-loss diets. Accordingly, four participants (14.8%) had a history of dropout of two diets, and five participants (18.5%) had a history of dropout of weight-loss diets three times. Furthermore, nine participants (33%) were single, and 18 (66.7%) were married. The reasons for going on weight-loss diets included dissatisfaction with body shape (66.6%), suffering from diseases (14.8%), and improvement of health status (18.5%) (Table 1).

**Table 1:** Demographic characteristics of participants

Characteristics	N (%)
**Mean Age (y ± SD)**	33/4 ± 8.4
**Female**	25 (92.6%)
**Male**	2 (7.4%)
**Educational level**	
• Below diploma	14 (51.8)
• Diploma level	9 (33.3)
• Academic level	4 (14.8)
**Body Mass Index**	
• Overweight	12 (44.4)
• Obesity	15 (55.5)
**Reason for diet for weight loss**	
• Body shape dissatisfaction	18 (66.6%)
• Improve health status	4 (14.8%)
• Diseases (hyperlipidemia, hypertension, etc.)	5 (18.5%)

Tables 2 and 3 describe quantitative interview information, such as who prescribed or recommended the diet for them. And what their diet included and the number of themes, and their repetition. (Tables 2,3).

**Table 2:** Sources of received diet among the participants and type of diet

Sources of received diet	N	Frequency	Diet type	
Nutritionist	17	63	Scientific diet	• Low -calorie diet • Low fat diet
Self- diet	5	18/5	Non- scientific diet	• Diet with 400–500 daily calories, • Only fruit-eating, • Only water drinking, • Complete removal of starch in daily diet
Friend recommendation	2	7.4		
Internet web page information	3	11.1

**Table 3:** Frequency distribution of each theme for dropout of diet

Themes	N	Percent
Theme 1: Individual reasons for dropout of diet	17	63
Theme 2: Family and social reasons for dropout of diet	14	51.9
Theme 3: Diet characteristics reasons for dropout of diet	9	33.3

After completing 27 interviews, 391 initial codes on dietary problems and reasons for dropout were extracted. Eventually, 181 codes were obtained, which were in the first ten categories. Subsequently, by merging the codes with similar concepts, approximately 28 final codes were extracted that were classified into 15 categories and three general themes (Tables 4,5,6).

**Table 4:** Individual reasons for dropout of diet

Them	Categories	Codes
Individual reasons for dropout of diet	Misunderstanding of diet	The idea that we can maintain weight without a diet
		Imagine losing weight too fast
	Not having enough motivation	lack of motivation
	Conditions of stress and hormonal disorder	Stress and hormonal disorder
		Taking hormonal drugs
	Feel harm to health	Having a weak state
		Having headaches
		Skin deterioration
		Having a burning stomach
		Nails become brittle
	Lack of mental and psychological preparation	Non- Helping your spouse get a diet
		Comment by friends about facial aging
		Lack of companionship to maintain a diet.
	Personal taste	Dislike diet foods

**Table 5:** Family and social reasons for dropout of diet

Them	Categories	Codes
**Family and social reasons for dropout diet**	Social problem	Busy and problems at work and school
	Family problem	Not accompanying family and friends

**Table 6:** Diet characteristics reasons for dropout of diet

Them	Categories	Code
Diet characteristics reasons for dropout of diet	Ineffective of diet	No weight loss
	Expensive of diet food and dietary supplement	Expensive food for diet
		Expensive weight loss supplements
	Unavailability of food	The unavailability of a particular food
		The unavailability of food in a different situation
	Unscientific and unconventional diets	Special diet
		Very limitation of food in the diet
	Feeling wrong about the diet	Not paying attention to the strong tendency of a particular food
	Non- palatable diet food	Not paying attention to individual un- like food

*Since some participants mentioned several reasons for diet dropout, some of them were put in separate categories that were concurrently relevant to two different themes

The first theme for diet dropout included personal reasons, categorized into six parts: diet misunderstanding, lack of motivation, stress and hormonal impairment, having the feel of “being harmful to health”, lack of mental and psychological preparation, and personal taste. Then, 17 codes were generated from the categorized subsets. Further details on the codes and examples have been presented in Table 4.

The second theme included familial and social reasons divided into two categories and two codes. In this context, two crucial codes were “being busy and having problems at work and school” and “lack of cooperation on the part of family and friends”. Further details on the codes and examples have been shown in Table 5.

The third theme was related to diet characteristics categorized into six parts: the diet’s ineffectiveness, expensiveness of diet food and dietary supplements, unavailability of food, unscientific and unconventional diets, feeling bad about the diet, and unpalatable diet food. Further details on the codes and examples have been presented in Table 6.

### Theme 1. Personal reasons for dropout of diet

The first theme was personal reasons for diet dropout, categorized into six parts: diet misunderstanding, lack of motivation, stress and hormonal impairment, feeling of “being harmful to health”, lack of mental and psychological preparation, and personal taste. Then, 17 codes were generated from the categorized subsets. What follows includes the details of the codes and examples.

Among the participants, the term “dropout” was used to describe withdrawal from dieting, such that overeating might occur. In other words, “premature termination of diet and gradual overeating” was often used to describe this phenomenon. In this theme, the reasons for the dropout of diet were explained.

Misunderstanding of diet: In this sub-theme, there were several concepts such as “the idea that we can maintain weight without going on a diet” and “imagination of quick weight loss”. Regarding the maintenance of weight without going on a diet, one of the participants stated:

“*After losing weight, I thought I no longer needed to be on a diet”* (participant No. 3).

#### Not having enough motivation

In this sub-theme, one crucial concept was “lack of motivation”. One of the participants said:

“*I didn't adhere to my diet at a party. We always had a party. Repeating this made me lose my diet, and my appetite increased again. So, I could no longer continue my diet program. The most important factor in my diet dropout was not motivated”* (participant No. 8).

#### Stress and hormonal disorder

In this sub-theme, there were several concepts such as “stress and hormonal disorder” and “taking hormonal drugs”. In this context, two participants maintained:

“*I experienced hormonal imbalance and weight gain a few years ago. I was offered a diet. I adhered to the diet for seven or eight months, and I went to a gym, as well. I experienced a good weight loss, and I was delighted. However, due to stress conditions and thyroid hormone problems, I regained my weight. This was repeated two or three years later … ”* (Participant No. 11).

“ … *After my weight was fixed, the doctor gave me a maintenance diet, but I neglected it. Besides, because of hormonal problems, my weight increased rapidly. I did not go to the doctor again to pursue this problem. I was also involved in depression. My appetite increased because of the hormonal disturbance and consumption of anti-depression drugs … ’* (Participant No. 5).

#### Having the feeling of “being harmful to health

In this sub-theme, there were several concepts such as “having headaches,” “having stomach burning,” and “having brittle nails.” In this regard, eight participants said:

“ … *The problem was that I was weak in the early days, and it was hard for me. I became very thin during the diet, but I had severe headaches, and my face had deteriorated. So, I dropped out of my diet. The problem with my diet was that it had some effects on my skin, and it didn't affect my body because I exercised. Because of my stubborn diet, my face and skin were flaky. I had hair loss and stomach burning. As a result of this diet, I had severe hair loss and had a lot of physical weakness, and my nails were broken. I called them and said I had hair loss, but they said it was OK … ”* (Participant No. 1).

#### Lack of mental and psychological preparation

In this sub-theme, there were several concepts such as “spouse’s lack of cooperation for adherence to the diet,” “friends’ comments about facial aging,” and “lack of companionship to maintain a diet.” In this respect, six participants mentioned:

“ … *I dropped out of the diet because my wife said that my face was shrinking and that I was getting old and broken. I lost a little weight, but weight loss affected my face a lot, and people around me kept saying that I looked too old. My diet didn't cause any problems, and it was perfect. Most troubles were related to those around us who insisted on eating wherever we went...”* (Participant No. 19).

#### Personal taste

In this sub-theme, there were several concepts such as “dislike for diet food,” “great interest in certain food items,” “high tendency towards sweets,” “lack of diet based on one’s interests and appetite,” and “too many restrictions in the diet.” In this regard, five participants said:

“ … *The problem with my diet was that there were lots of food items that I didn't like. I was fond of pizza and soda and couldn't remove them from my diet. During the diet, I found myself craving a lot of sweets. All my favorite food items were eliminated from the diet, and the amount of food was insufficient for my appetite. It was challenging for me to restrict starchy food and fat in the diet, and I didn't feel good about it...”* (Participant No. 2).

### Theme 2. Familial and social reasons for dropout of diet

The study findings reflected the relationship between personals as well as their backgrounds and social and familial factors. This theme included two categories and two codes. The details of the codes and examples have been presented below.

#### Social problems

This sub-theme consisted of one concept; i.e., “being busy and having problems at work and school.” In this regard, two participants said:

“ … *My main problem was my work. I became hungry during the nights because I was on shift work and had woken up in the morning. I didn't know what to do, and I was starving. Due to the cramped university classes and lack of time, I couldn't adhere to my diet schedule. Besides, I couldn't exercise … ”* (Participant No. 6).

#### Familial problems

This sub-theme consisted of one concept; i.e., “lack of cooperation on the part of family and friends.” In this context, three participants maintained:

“ … *My daughter got married. We had lots of parties. My son-in-law’s family insisted on eating every time. After that, I gradually dropped out of my diet that was going on well … ”* (Participant No. 7).

### Theme 3. Reasons related to diet characteristics

The third theme included the reasons related to diet characteristics and was categorized into six parts: the ineffectiveness of diet, the expensiveness of diet food and dietary supplements, unavailability of food, unscientific and unconventional diets, and feeling bad about the diet, and unpalatable diet food. Further details about the codes and examples have been provided below:

#### Ineffectiveness of diet

This sub-theme consisted of one concept; i.e., “no weight loss.” Two participants said:

“ … *I was very motivated for the first couple of months. However, when my weight didn’t change, I lost my motivation … ” (*Participant No. 5).

#### Expensive diet food and dietary supplements

This sub-theme included two concepts, namely “expensive diet food” and “expensive weight loss supplements.” In this regard, two participants stated:

“ … *The problem with my diet was that it wasn't economical, and it was very difficult to get diet food. So, I couldn’t adapt to the diet, and I lost my motivation gradually. Weight-loss supplements are generally expensive, and they are not worthy for long-term usage* … ” (Participant No. 13).

#### Unavailability of food

This sub-theme consisted of two concepts, namely “unavailability of certain food items” and “unavailability of food in different situations.” In this respect, two participants said:

“ … *The problem with my diet was that it was challenging to get food. So, I couldn’t adapt to the diet, gradually left, and did not attend the therapy sessions. My lunch and dinner meals were almost just meat, poultry, and grilled fish in my diet. Normally, this type of food was not always available to me since I was at work or because of attending parties and family gatherings or some other restrictions … ”* (Participant No. 18).

#### Unscientific and unconventional diets

This sub-theme consisted of two concepts, namely “unavailability of certain food items” and “unavailability of food in different situations.” In this regard, five participants maintained:

“ … *I used different diets. Some were simple diets that had everything, and some were starchy and sugar-free. However, some were special diets that only had coffee or bananas. I tried different diets with temporary effects. I had no rice in my diet for the first month. I only had lentils or egg white for breakfast. Lunch and dinner included chicken breast or steamed fish and vegetables … ”* (Participant No. 22).

#### Feeling bad about the diet

This sub-theme included one concept; i.e., “not paying attention to the strong tendency towards certain food items.” In this regard, two participants mentioned:

“ … *At that time, I had a strong tendency towards chocolate and cocoa, which was limited, and my blood pressure dropped. In addition, I had headaches and felt weak almost all the time. I was very bothered and did not feel well* … ” (Participant No. 24).

#### Unpalatable diet food

This sub-theme consisted of one concept; i.e., “not paying attention to personal unpalatable food items.” In this context, two participants said:

“ … *My previous diet consistently made me eat beans or lentils, cooked vegetables, coconut, chicken, and boiled fish, which I didn't like at all. There were lots of food items in my diet that I didn't like … ”* (Participants No. 14 and 10).

## Discussion

Dropout of diet is one of the major problems in obesity management. Qualitative studies may have a suitable design to address this problem. In the current study, 28 final codes were extracted and classified into 15 categories. Finally, three general themes were obtained: personal reasons for dropout of diet, familial and social reasons, and reasons related to diet characteristics.

The study results indicated the importance of personal reasons for diet dropout. This theme consisted of six subcategories, including diet misunderstanding, lack of motivation, stress and hormonal disorder, having the feel of “being harmful to health”, lack of mental and psychological preparation, and personal taste. Similarly, Anderson emphasized that patient-related factors could influence the maintenance of diets [18]. Lack of readiness and mental support was also one of the personal factors that could affect diet dropouts. In the same line, Fidelix et al. revealed a significant relationship between diet dropout and depression, anxiety, body dissatisfaction, emotional role, and mental health as psychological parameters. Furthermore, Fassino et al. indicated that the combination of low self-management and low participation could affect the dropout of treatments [16]. Alice et al. also disclosed that depression, stress, strong body shape concern, previous weight-loss attempts, and being unemployed were essential factors in the continuation or dropout of the treatment process [19].

The findings of the present qualitative analysis suggested that weight-loss diet dropout could be attributed to familial and social reasons. This theme included two sub-themes: being busy and having problems at work and school and having family members and friends who did not accompany them in the weight-loss procedure. A previous study also demonstrated that one of the most essential factors in diet dropout was the lack of support from family members and friends [20, 21]. The results of a study by Bruges et al. In a systematic review also showed that there should be new insights for physicians specializing in obesity. They must be careful in designing a lifestyle intervention program. Barriers to behavior change should be removed early in treatment and lifestyle intervention based on it. First, the negative mood and unrealistic expectations of weight loss should be discussed.

The recent study also indicated that one of the main factors in dropout rates in obesity managmants is family factors. Thus, clinicians and dietitians to improve diet consultation and decrease dropout rate need to more attention supportive family environment in weight mangmant program [22].

Determining the characteristics that contribute to dropout in weight management program is crucial for identifying individuals who are at high risk of dropping out. In this area, psychological health issues such as depression, anxiety, low self-esteem, and body dissatisfaction may have an impact on the result of a weight-loss program and the dropout rate [23–25]. The current study also showed that mental and psychological preparation could influence in droup out rate. Determining psychological health in weight management and weight dropout is an important issue for future research.

The results of the current qualitative research showed that weight-loss diet dropout could be attributed to diet characteristics. This theme had six subcategories: ineffectiveness of diet, expensive diet food and dietary supplements, unavailability of food items, unscientific and unconventional diets, feeling bad about the diet, and unpalatable diet food and People’s inability to provide nutrients and low in calories is an important factor in dropout of a weight loss diet. Ferdosian et al. reported that 42% of the participants suffered from low-calorie diets as the most crucial issue with improvement in their weight-loss programs. On the other hand, 81% of the participants stated that concordance of the diet with daily work schedules and decrease of starch and sweets were the factors that contributed to the success and maintenance of the weight-loss program. The expensiveness of food or dietary supplements was also one of the most important factors in that study [26]. In the same line, Ahnis et al. revealed that expensive diets were associated with diet dropout. A previous study also indicated that economic status could determine the success rate or dropout of diets [27–29].

The effect of “hormonal disorders” on withdrawal from diet is not directly mentioned in any of the studied sources, but some studies have suggested the effect of diseases on withdrawal from treatment, which can be classified as hormonal disorders [26, 30, 31].. However, the study and recognition of hormonal disorders independently as one of the reasons for withdrawal from the treatment process in obese and overweight patients, has been done in this study for the first time.

Feeling unhealthy due to diet was one of the reasons for excluding the diet in this study. The results of the study of Ahnis et al. Show that there is a relationship between “getting sick” and withdrawing from the treatment regimen process (31). Which is consistent with the results of studies by Ferdowsian et al. And Anderson et al. [26, 31].

### Application of findings

The current study findings can be used to develop principles and guidelines for prescribing diets for patients and resetting diets for patients who have withdrawn from their diets. Moreover, universities and academic institutions are recommended to explore the causes of dieting and obesity, which endanger the health of society. They must also find solutions to remove social barriers and educate people about familial barriers. This will eventually help people establish a healthy lifestyle. The strength of this study is to provide a deep understanding of how people drop out in a weight loss diet, which is the actual reality for nutritionists. Also, the limitation of this research was that none of the patients was evaluated in a private clinic.

## Conclusion

Considering the increasing prevalence of obesity and the high rates of diet dropouts, a deep understanding of the reasons for diet dropouts may be helpful. In the present study, the reasons for diet dropout were identified in three levels, namely personal reasons for dropout of diet, familial and social reasons, and reasons related to diet characteristics. Hence, clinicians are recommended to pay more attention to the complexity of weight management and weight-loss maintenance. Moreover, paying attention to personalized diets, characteristics of diets, and familial and social support is suggested for formulating an efficient weight management diet.

## Data Availability

The datasets produced and analyzed during the present study are not publicly accessible due to participant confidentiality, but are obtainable from the corresponding author on reasonable request.

## References

[1] Heideman W, Rongen F, Bolleurs C, Govers E, Kroeze W, Steenhuis I (2019). Facilitators and barriers to a dietitian-implemented blended care weight-loss intervention (SMART size): a qualitative study. J Hum Nutr Diet.

[2] Olivan-Blázquez B, Montero-Marin J, García-Toro M, Vicens-Pons E, Serrano-Ripoll M, Castro-Gracia A (2018). Facilitators and barriers to modifying dietary and hygiene behaviours as adjuvant treatment in patients with depression in primary care: a qualitative study. BMC Psychiatry.

[3] Fallahzadeh H, Saadati H, Keyghobadi N (2017). Estimating the prevalence and trends of obesity in Iran populations from 2000 to 2011: a meta-analysis study. SSU_J.

[4] Abdelaal M, le Roux CW, Docherty NG (2017). Morbidity and mortality associated with obesity. Ann Transl Med.

[5] Vuik S, Lerouge A, Guillemette Y, Feigl A, Aldea A (2019). The economic burden of obesity. The economic burden of obesity.

[6] Rosnov DL, Roberts MC, Kessler ED, Pendley JS, Datto G, Hassink S (2011). Acceptability of weight-loss interventions among adolescents who are overweight and their caregivers. Child Health Care.

[7] Baygi F, Qorbani M, Dorosty A, Kelishadi R, Asayesh H, Rezapour A, Mohammadi Y, Mohammadi F (2013). Dietary predictors of childhood obesity in a representative sample of children in north east of Iran. Zhongguo dang dai er ke za zhi = Chinese journal of contemporary pediatrics.

[8] Latner JD, Ciao AC (2014). Weight-loss history as a predictor of obesity treatment outcome: prospective, long-term results from behavioral, group self-help treatment. J Health Psychol.

[9] Lobstein T, Jackson-Leach R, Moodie ML, Hall KD, Gortmaker SL, Swinburn BA, James WPT, Wang Y, McPherson K (2015). Child and adolescent obesity: part of a bigger picture. Lancet.

[10] Wilfley DE, Tibbs TL, Van Buren D, Reach KP, Walker MS, Epstein LH (2007). Lifestyle interventions in the treatment of childhood overweight: a meta-analytic review of randomized controlled trials. Health Psychol.

[11] Alexander E, Tseng E, Durkin N, Jerome G, Dalcin A, Appel L (2018). Factors associated with early dropout in an employer-based commercial weight-loss program. Obes Sci Pract.

[12] Ortner Hadžiabdić M, Mucalo I, Hrabač P, Matić T, Rahelić D, Božikov V (2015). Factors predictive of dropout and weight loss success in weight management of obese patients. J Hum Nutr Diet.

[13] Lau DC, Douketis JD, Morrison KM, Hramiak IM, Sharma AM, Ur E (2007). 2006 Canadian clinical practice guidelines on the management and prevention of obesity in adults and children [summary]. CMAJ..

[14] Aurini JD, Heath M, Howells S (2016). The how to of qualitative research: Strategies for executing high quality projects.

[15] Naderifar M, Goli H, Ghaljaie F (2017). Snowball sampling: a purposeful method of sampling in qualitative research. Strides Dev Med Educ.

[16] Mazaheri M, Eriksson LE, Heikkilä K, Nasrabadi AN, Ekman SL, Sunvisson H (2013). Experiences of living with dementia: qualitative content analysis of semi-structured interviews. J Clin Nurs.

[17] Tabatabaee A, Hasani P, Mortazavi H, Tabatabaeichehr M (2013). Strategies to enhance rigor in qualitative research. J North Khorasan Univ Med Sci.

[18] Forero R, Nahidi S, De Costa J, Mohsin M, Fitzgerald G, Gibson N, McCarthy S, Aboagye-Sarfo P (2018). Application of four-dimension criteria to assess rigour of qualitative research in emergency medicine. BMC Health Serv Res.

[19] Anderson JW, Konz EC, Frederich RC, Wood CL (2001). Long-term weight-loss maintenance: a meta-analysis of US studies. Am J Clin Nutr.

[20] Fassino S, Pierò A, Tomba E, Abbate-Daga G (2009). Factors associated with dropout from treatment for eating disorders: a comprehensive literature review. BMC Psychiatry.

[21] Burgess E, Hassmén P, Pumpa KL (2017). Determinants of adherence to lifestyle intervention in adults with obesity: a systematic review. Clin Obesity.

[22] Park J, Woo S, Ju Y-S, Seo Y-G, Lim H-J, Kim Y-M, Noh HM, Lee HJ, Park SI, Park KH (2020). Factors associated with dropout in a lifestyle modification program for weight management in children and adolescents. Obes Res Clin Pract.

[23] Sawamoto R, Nozaki T, Furukawa T, Tanahashi T, Morita C, Hata T, Komaki G, Sudo N (2016). Predictors of dropout by female obese patients treated with a group cognitive behavioral therapy to promote weight loss. Obes Facts.

[24] Lazzeretti L, Rotella F, Pala L, Rotella CM (2015). Assessment of psychological predictors of weight loss: how and what for?. World J Psychiatry.

[25] Leung AW, Chan RS, Sea MM, Woo J (2017). An overview of factors associated with adherence to lifestyle modification programs for weight management in adults. Int J Environ Res Public Health.

[26] Sahar F, Kimiagar SM (2010). Factors affecting the reduction of body profile in women under weight loss. J Kerman Univ Med Sci.

[27] Mutsaerts M, Kuchenbecker W, Mol B, Land J, Hoek A (2013). Dropout is a problem in lifestyle intervention programs for overweight and obese infertile women: a systematic review. Hum Reprod.

[28] Gorecki MC, Feinglass JM, Binns HJ (2019). Characteristics associated with successful weight management in youth with obesity. J Pediatr.

[29] Bristow C, Meurer C, Simmonds J, Snell T (2020). Anti-obesity public health messages and risk factors for disordered eating: a systematic review. Health Promot Int.

[30] Andersson I, Rössner S (1997). Weight development, drop-out pattern and changes in obesity-related risk factors after two years treatment of obese men. Int J Obes Relat Metab Disord.

[31] Ahnis A, Riedl A, Figura A, Steinhagen-Thiessen E, Liebl ME, Klapp BF. Psychological and sociodemographic predictors of premature discontinuation of a 1-year multimodal outpatient weight-reduction program: an attrition analysis. J Patient Preference Adherence. 2012:67165–77. 10.2147/PPA.S28122. 10.2147/PPA.S28022 PMC3307662 22442628

